# Atypical scrotal edema – Case report

**DOI:** 10.1177/2050313X241260982

**Published:** 2024-06-20

**Authors:** Ariana Nateghi, Rose Noël, Andréanne Waddell, Mylène Veilleux

**Affiliations:** Department of Dermatology, University of Sherbrooke, Sherbrooke, Canada

**Keywords:** Tropical disease, Filariasis, Lymphedema

## Abstract

Lymphatic filariasis is an endemic parasitic infection in 72 countries. It is caused by a filarial worm transmitted through mosquito bites. Acute nonspecific symptoms can occur, such as fever and edematous inflammatory plaques, while its chronic state is commonly characterized by lymphedema.

## Case report

**Figure 1.**
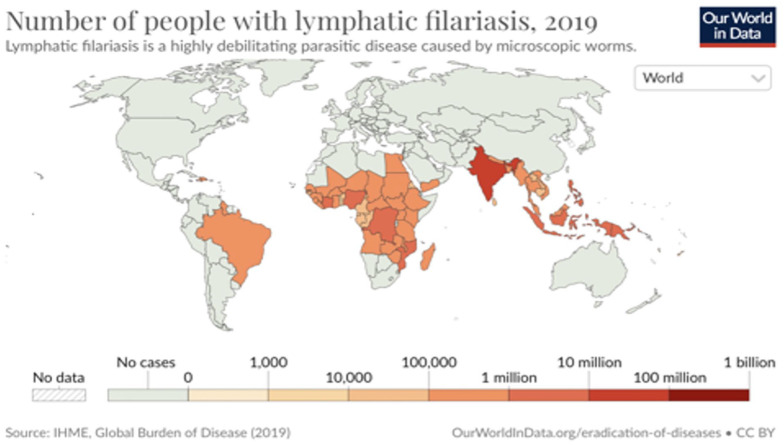
Severe scrotal and suprapubic lymphedema.

**Figure 2.**
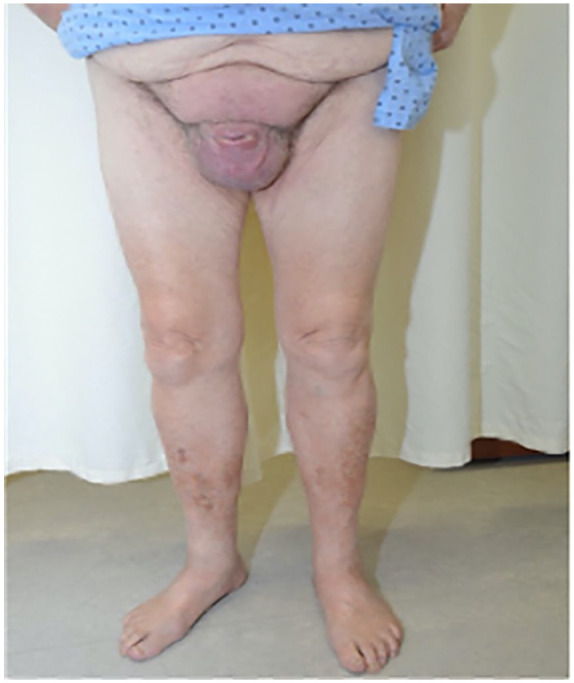
Number of people with lymphatic filariasis, 2019.^3^

This case presents a 73-year-old patient, consulting for scrotal and suprapubic lymphedema, progressing over 8 years (Figure 1). The patient’s lower limbs were uninvolved, and did not present any systemic symptoms. Skin biopsies showed nonspecific changes, without granulomas. A full blood count indicated no eosinophilia and normal levels of fecal calprotectin. A computed tomography scan of the pelvis and abdomen showed no signs of obstruction. Finally, two filariasis serologies returned positive. The diagnosis of lymphatic filariasis (LF) was made in 2020 (Figure 2).^1^

The patient visited Barbados, a Caribbean country, in the early 1970s, when *Wuchereria bancrofti* (WB) was endemic in the region.^2^ He had reported edema of the right lower limb in 1977. In 1979, he was diagnosed with a hydrocele. He was treated in 1980, with suspicion of an orchiepididymitis. Subsequently, the patient was seen in a specialized clinic where it was agreed that anti-parasitic treatment would not be advisable given the diagnostic delay. He is now waiting for a surgical correction for his lymphoedema.

LF is transmitted by a female mosquito bite. *WB, Brugia malayi*, and *Brugia timori* are the three nematode worms responsible for LF, WB being responsible for 90% of LF cases.^3^ To contract LF, repeated mosquito bites over several months are necessary. As a result, travelers rarely develop chronic manifestations; however, literature reports few cases.^4^

While most infected are asymptomatic, the lymphatic damage caused by Mf leads to the development of acute lymphangitis stage. This stage is marked by fever, rash, erythematous streaks on arms, painful lymph nodes, orchitis, and epididymoorchitis.^3^ Later, dermatolymphangioadenitis (ADLA) may cause secondary bacterial infections like cellulitis and abscess formation. In the chronic stage, patients may experience edema, hydrocele, and rarely, renal impairments such as nephrotic syndrome.^3^ If untreated, it can progress to elephantiasis. Filarial hydroceles are the most common chronic manifestation in men.^3^

Different diagnosis methods are used.^3^ Although serological tests for filarial antibodies cannot differentiate between active and chronic infection, they are useful for detecting infection in travelers from previously endemic areas, as in our patient.

In conclusion, the Global Programme to Eliminate Lymphatic Filariasis has made significant strides in reducing new cases worldwide by administering over 9 billion chemoprophylactic treatments, resulting in a 74% decrease in new cases.^5^ With 51.4 million people still affected today,^5^ it underscores the critical need to examine LF in the differential diagnosis of chronic genital lymphedema. Considering historical disease prevalence is important when assessing travelers for filariasis manifestations, along with the current endemic status. However, for chronic manifestations, as in our case, the importance of evaluating the destination’s endemic status at travel time is trivial. By incorporating these considerations into clinical practice, we can enhance the early detection and management of this debilitating condition.
